# Healthcare professionals' perspectives on diagnostic delay in rare cancers and options for patient support: a qualitative interview study

**DOI:** 10.3389/fpsyg.2026.1945741

**Published:** 2026-10-09

**Authors:** Femke Krause, Liza A. Hoveling, Rosalie Wilbrink, Denise Abbel, Kristel M. van Asselt, Filip de Vos, Saskia F. A. Duijts

**Affiliations:** 1 Department of Research and Development, Netherlands Comprehensive Cancer Organisation (Integraal Kankercentrum Nederland), Utrecht, Netherlands; 2 Department of General Practice and Nursing Science, UMC Utrecht, Utrecht, Netherlands; 3 Department of Medical Oncology, University Medical Center Utrecht, Utrecht, Netherlands; 4 Department of Medical Psychology, Amsterdam University Medical Center, Location Vrije Universiteit, Amsterdam, Netherlands; 5 Cancer Treatment and Quality of Life, Cancer Center Amsterdam, Amsterdam, Netherlands

**Keywords:** delay, diagnostic trajectory, healthcare professionals, oncology, rare cancer, supportive care

## Abstract

**Purpose:**

Delayed diagnosis in rare cancers can lead to disease progression, limited treatment options, and psychological distress, resulting in poorer quality of life and disability. We explored healthcare professionals' (HCPs) perceptions of diagnostic delay in rare cancers and options for patient support.

**Materials and methods:**

Semi-structured interviews were conducted with 20 HCPs involved in rare cancer care. Interview topics covered diagnostic challenges, causes of delays, consequences for patients, and support options. Data were analyzed thematically.

**Results:**

Three main themes emerged: (1) *Time, intuition, and a safety-net fill the gap when signs are present, but not obvious*. HCPs balance clinical intuition with safety-netting to avoid mis-, under-, or overdiagnosis when symptoms are vague, (2) *Balancing speed and care in a fast-paced, complex diagnostic phase*. Once cancer is suspected, the trajectory accelerates, yet systemic delays persist, and (3) *Striving for precision: the paradox of reaching a diagnosis*. While HCPs pursue diagnostic clarity, uncertainty often causes patients more distress than the diagnosis.

**Conclusion:**

HCPs manage the diagnostic trajectory of rare cancers through clinical judgment, safety-netting, and clear communication. As systemic delays and patient distress remain, early psychosocial support may help. Future research should define diagnostic delay, assess its impact, and identify support options.

## Introduction

Diagnosing rare cancers is often a complex process due to their low incidence and challenges such as limited or inadequate tumor samples and variations in pathology assessment (Pillai and Jayasree, 2017; Padilla et al., 2024). This complexity is further exacerbated by, among others, heterogeneous clinical presentations, limited experience among healthcare professionals (HCPs), e.g., general practitioners (GP) and medical specialists, and a lack of public awareness (Padilla et al., 2024). These factors may contribute to delayed diagnosis, frequent misdiagnoses, and limited access to specialized care and appropriate treatments in patients with a rare cancer. Additionally, HCPs may be confronted with clinical uncertainty during decision-making (Pillai and Jayasree, 2017).

Rare cancers are characterized by an incidence rate of less than 6 per 100,000 people annually, as defined by the Surveillance of Rare Cancers in Europe (Gatta et al., 2011). Approximately 20% of cancer diagnoses in concerns a rare cancer, with more than 20,000 people receiving a rare cancer diagnosis annually in the Netherlands (Blaauwgeers et al., 2018). Moreover, 5-year survival rates of rare cancers have not increased by the same rate as common cancers, with rare cancers increasing 50% to 56% from 1995 to 2016, compared to 59% to 72% for common cancers in the same period (De Heus et al., 2022). Further, patients with a rare cancer often experience psychological distress and lower quality of life (QoL), compared to patients with a common cancer. These differences are related to diagnostic uncertainty, unpredictability of disease progression, and limited treatment and rehabilitation options (De Heus et al., 2023). Such experiences may undermine their trust in the healthcare system and in HCPs (Simons et al., 2025), while at the same time they report an overall lack of support throughout their care trajectory (De Heus et al., 2023).

The diagnostic trajectory of cancer commonly consists of sequential time intervals from initial symptoms to diagnosis, namely: Patient Interval (IP; i.e., time from first symptoms to first primary care visit); Primary Care Interval (IPC; i.e., time from first GP consultation to referral to medical specialist); and Secondary Care Interval (ISC; i.e., time between medical specialist referral to diagnosis; Van Erp, 2021). While the IP can be influenced by patient factors, such as awareness of symptoms, recognizing their severity, and understanding the necessity of seeking medical attention, HCPs play a pivotal role in shaping the overall diagnostic process across the IPC and ISC (Moore et al., 2024). That is, in primary care, GPs are often the first healthcare contact for patients presenting with potential cancer-related symptoms (Green et al., 2015). However, due to their rarity and often non-specific symptoms, rare cancers are frequently not recognized by GPs during initial consultations (IKNL, 2023). Diagnostic uncertainty, and the risk of attributing symptoms to more prevalent conditions, represent a challenge in general practice and constitutes a key barrier to the timely identification of rare cancers (Moore et al., 2024). When patients are referred to medical specialists, the diagnostic process is often obstructed by challenges such as time limitations, insufficient disease-specific knowledge, and broader organizational issues, such as clarity about the designated center of expertise for a specific rare cancer type (Adams et al., 2024).

The diagnostic trajectory follows standard methods, including physical exams, biopsies, imaging, and molecular analyses (Christyani et al., 2024). Althought these conventional diagnostics work well for common cancers, they regularly overlook rare cancers because these often present with similar physical traits. While molecular analyses have shown potential for improving early detection of rare cancers (Christyani et al., 2024), patients may still face additional obstacles in secondary care. Histopathological examination is essential in diagnosing rare cancers, however, has yet been shown to experience error in up to 40% of cases due to inadequate tissue samples (Pillai and Jayasree, 2017). Such diagnostic errors may result in misdirected care pathways (Padilla et al., 2024). In one study, 32% of patients with a rare cancer reported an initial misdiagnosis, visiting multiple hospitals before receiving the correct diagnosis. Patients with a common cancer, however, are more likely to be diagnosed within a single center (Padilla et al., 2024).

Timely access to optimal care remains challenging for patients with a rare cancer and may negatively contribute to patient experiences (Padilla et al., 2024). As such, supportive care during the diagnostic trajectory is essential (Berman et al., 2020). Early integration of supportive care has been found to help manage emotional distress, provide better guidance through the healthcare system, and enhance patient satisfaction and rehabilitation outcomes (Haroen et al., 2025). As HCPs have an important role in addressing emotional and informational needs in patients navigating this uncertain and often distressing phase, their perspectives regarding diagnostic difficulties and related support are crucial.

Therefore, the aim of this study was to explore HCPs' perspectives on diagnostic delay in rare cancers and to identify options for patient support during the diagnostic trajectory.

## Materials and methods

### Study design

A qualitative exploratory design was used, through semi-structured interviews with HCPs, to gain an in-depth understanding of the complexities regarding the diagnostic trajectory of rare cancers. Ethical approval was obtained from the Medical Ethics Committee of Amsterdam UMC, which concluded that the Medical Research Involving Human Subjects Act (WMO) did not apply (2024.1083).

### Study sample and recruitment

HCPs, such as GPs, pathologists, and medical oncologists, were eligible for participation if they were involved in, or have knowledge about, the diagnostic trajectory of patients with a rare cancer. HCPs were recruited through the involved researchers' networks, the Dutch Rare Cancer Platform (DRCP), and social media. Additionally, snowball sampling was used to further expand the participant pool. Prior to inclusion, potential participants received a short questionnaire to provide their sociodemographic data and informed consent. Upon return of the completed and signed documents, eligibility was checked, after which an invite was sent for an interview. Recruitment continued until data saturation was reached.

### Interview guide and data collection

The research team consisted of members with diverse backgrounds including two health sciences master students (FK, RW), three researchers in (psychosociol) oncology without clinical work experiences (SD, LH, DA), a general practitioner (KvA) and a medical oncologist (FdV). The team as a whole has extensive experience in qualitative research.

The interview guide was developed based on the literature and the expertise of the project team. Topics included diagnostic challenges, factors contributing to delay, consequences of a delayed diagnosis, potential improvements, and patient support (see supplement Interviewguide). The interview guide was evaluated and adjusted by FK, LH and SD, and a trial interview was conducted in order to make final adjustments before the first interview. The trial interview was excluded from data analysis. Interviews were conducted between March and May 2025, via Microsoft Teams. All interviews were recorded with audio equipment, transcribed verbatim and anonymized. A second member of the team (RW, LH or SD) was present during the interviews to operate the recording equipment, make notes, and reflect on the interviews afterwards.

### Data analysis

Descriptive statistics were used to illustrate participant characteristics, based on data from the questionnaires. Thematic analysis of interview data followed the six-phase approach described by Braun and Clarke (Braun and Clarke, 2006). Three interviews were coded by two independent researchers (FK and RW) to establish and ensure reliability of the coding procedure. Varying interpretations were discussed to explore reasons for disagreement, and to reach consensus. The remaining interviews were coded by a single researcher (FK). Using the constant comparative method, codes were analyzed within and across interviews to identify patterns, themes, and relationships in the data using ATLAS.ti. This method involves continuously comparing data throughout the coding process to refine themes and deepen understanding. Themes were discussed among the researchers until consensus was reached regarding the main themes. Illustrative quotes were reviewed by a native speaker to ensure linguistic accuracy.

## Results

### Sample characteristics

Twenty HCPs, ranging in age from 36 to 72 years (mean: 48; SD 10), were interviewed. Of the participants, nine were men and eleven were women. The HCPs occupied diverse roles during the diagnostic trajectory, covering both primary and secondary levels of care, such as GPs and medical oncologists. HCPs were employed at different hospital types. For an overview of characteristics, see Table 1.

**Table 1:** Sample characteristics.

Characteristics	Participants (*n* = 20); *n* (%)
Gender	
Male	9 (45)
Female	11 (55)
Age in years, mean (SD)	48.35 (9.95)
Profession	
General practitioner	4 (20)
Pathologist	1 (5)
Oncologist	6 (30)
Oncological surgeon	2 (10)
Radiotherapist	1 (5)
Ophthalmologist	1 (5)
Pulmonologist	2 (10)
Nuclear medicine physician	1 (5)
Dermatologist	1 (5)
Nurse practitioner	1 (5)
Years of employment, mean (SD)	14.4 (10.07)
Type of hospital	
Academic	15 (75)
Top clinical	1 (5)
General practice	4 (20)

### Themes

Three themes emerged from the data, reflecting HCPs' perspectives on diagnostic delay in rare cancers and their ideas regarding patient support: (1) *Time, intuition, and a safety net fill the gap when signs are present, but not obvious*, (2) *Balancing speed and care in a fast-paced, complex diagnostic phase*, and (3) *Striving for precision: the paradox of reaching a diagnosis*. All three themes are all related to challenges regarding navigation of competing diagnostic risks, including avoiding risk of over-diagnosis and system overload, balancing risk of missing or delaying diagnosis, and the risk of misdiagnosis. A complete overview of supporting quotes has been added as supplement.

## Time, intuition and a safety net fill the gap when signs are present, but not obvious

Interviewed HCPs stated that the diagnostic trajectory of some rare cancers begins with vague, non-specific, and slowly evolving symptoms, making early recognition extremely challenging. These symptoms are commonly associated with benign, everyday conditions, which can delay consideration of a more serious underlying cause such as a rare cancer. One HCP explained:

“*They are often very vague and difficult to pinpoint symptoms. Like a stuffy nose, for example, well, everyone gets that from time to time.”* (Male, 38 years old)

Another interviewee noted:

“*Let me emphasize that, in the early stages of symptoms in someone with a brain tumor, things are often very unclear, very vague, vague symptoms. People often think, ‘Oh yeah, a brain tumor always starts with headaches', but that's not the case. It can be very subtle things: somewhat forgetful, maybe burned out, overworked, depressed, which in hindsight, turn out to have been the first signs of a brain tumor.”* (Male, 49 years old)

Expanding on this point, another HCP highlighted how GPs' diagnostic reasoning is grounded in their familiarity with common, self-resolving illnesses:

“*The point is that general practioners are well trained in recognizing what is normal, what a normal flu looks like, what a normal cold looks like.”* (Male, 41 years old)

When patients present with vague symptoms in primary care, GPs often face a dilemma: while they may sense something is wrong, the signs are typically too inconclusive to justify immediate referral. One GP illustrated the underlying reasoning:

“*As a general practitioner, you are constantly assessing probabilities and risks, and rare diagnoses are always somewhere on your list, but usually very low. The prior probability is typically very small. So, before the patient even walks in, the likelihood is already low, and even after hearing the patient's story, it often remains low. I think it is good practice not to immediately pursue extensive diagnostics.”* (Female, 44 years old)

Follow-up consultations and monitoring subtle developments allow GPs to manage uncertainty, while weighing the risks of missing serious ilnessess against the downsides of overinvestigation and unnecessary referrals. This role as a “clinical gatekeeper,” navigating uncertainty while trying to avoid mis-, under-, and overdiagnosis, is recognized by HCPs in both primary and secondary care. Several participants acknowledged, however, that misdiagnosis is an inherent risk within this balancing act. As one participant reflected:

“*If we were to diagnose everyone very quickly and run extensive tests on everyone, we might miss fewer tumors, or perhaps none at all, but we'd also end up doing a lot of unnecessary things. This daily risk assessment is part of our profession, and inevidebly, the downside is that we are going to miss things as well.”* (Female, 46 years old)

When GPs do suspect that something may not be right, clear communication with the patient is seen as essential. One participant emphasized:

“*It's a balance between being honest, like saying, ‘this complaint or symptom doesn't sit well with me', or ‘I don't fully trust it', or ‘I'm a bit concerned', and at the same time not unnecessarily frightening the patient.”* (Female, 46 years old)

One HCP emphasized the importance of guiding patients through the diagnostic process by offering transparent and realistic communication, not just about outcomes, but about the steps involved and the uncertainties that may arise along the way:

“*Yes, I think clarity is crucial, really taking the time to explain why certain steps are taken. For patients, it can be very difficult to understand that, even in this day and age, we don't immediately know exactly what's going on or what the plan to diagnose and treat will be. I believe it's important to take the time to explain that clearly, be honest about current gaps in knowledge or what is still needed, and to provide a realistic timeline. Don't say, ‘We'll do a biopsy today and have the results tomorrow', if that's not the case, because we won't.”* (Female, 44 years old)

In these ambiguous cases, time is deliberately used as a diagnostic tool. Several GPs described how they allow the clinical picture to evolve over time before taking further steps. As one GP noted:

“*There is certainly a delay involved, which is partly intentional, as we consciously use time as a diagnostic tool.”* (Male, 41 years old)

To safely manage this intentional delay, safety-netting strategies are commonly employed:

“*I still quite often find myself giving what's called safety-netting advice; telling patients to return if their symptoms haven't resolved within three weeks.”* (Female, 44 years old)

However, safety-netting is not foolproof. Its effectiveness depends on multiple factors:

“*So no, sometimes it's still about following up on that kind of advice, and of course that depends both on how you communicate it, how the person takes it in, and also on who they are and how life unfolds.”* (Female, 44 years old)

In this gap between vague symptoms and definitive diagnosis, vigilance and intuition become crucial in recognizing and managing the risk of serious illnesses, such as rare cancers. Beyond structured protocols, GPs must rely on reflective judgment and tacit knowledge when the clinical picture remains unresolved. One HCP advised:

“*Continue to heed your ‘something-is-not-right' sense and remain vigilant as a physician: when the clinical picture does not fit, reflect and act accordingly. Stay alert if a patient returns several times without a clear explanation and intervene as needed.”* (Female, 36 years old)

## Balancing speed and care in a fast-paced complex, diagnostic phase

The GP plays a crucial role, acting as the primary point of access to specialized care. According to one HCP, the GP is considered the ‘main supplier' of the hospital, highlighting the responsibility they carry in ensuring patients are referred timely and effectively. This involves not only knowing how and where to refer patients, but also providing clear, relevant information to streamline the process. One HCP emphasized the importance of this role, stating:

“*No, that does fall under the responsibility of the general practitioner, to make a proper plan and ensure that the referral is appropriate.”* (Male, 41 years old)

However, even with appropriate referrals, the diagnostic phase for rare cancer remains challenging. Rare cancers often lack clear diagnostic pathways, which can complicate and delay the diagnostic phase. One key reason for this is the limited knowledge surrounding these cancers. As one HCP explained:

“*And so, it's inherently a more difficult population, because it's not a homogeneous group. We're dealing with gaps in our knowledge, and we have to deal with knowledge of lower quality.”* (Male, 54 years old)

Uncertainty remains a challenge during the whole diagnostic trajectory. HCPs mentioned that this arises from limited knowledge, unpredictable disease courses, and varied patient presentations. Without clear guidelines, HCPs rely on clinical judgment and must adapt to each rare cancer case. As one HCP noted:

“*Sometimes you have to work with the tools you have and navigate the uncertainties, and that's sometimes the art of medicine: figuring out how to ensure as swift and smooth a diagnostic process as possible.”* (Female, 62 years old)

Once cancer is suspected, the diagnostic and referral process typically speed up significantly. As one HCP noted:

“*Look, the moment we - as general practitioners - think this might be cancer, things usually move quickly.”* (Female, 46 years old)

This acceleration is often more pronounced in academic hospitals, where rare cancers are identified more efficiently due to greater exposure and experience. One HCP explained:

“*If you work in an academic hospital and you see rare pathologies more often, you‘re able to recognize them more easily. And that's not at all a criticism of those who mainly see common conditions, they're often much better at spotting those presentations. But for rare cases, it can lead to delays. The likelihood of a disease influences how thorough your diagnostics are. But it's not just about seeing it, you have to recognize it in the patient's story.”* (Male, 43 years old)

Although the diagnostic process often accelerates once the patient enters secondary care, several HCPs noted that delays can still occur while waiting for specific diagnostic procedures. And according to many HCPs, the phase of diagnostic uncertainty for the patient is the worst:

“*People prefer to know sooner where they stand psychologically, so I notice that they struggle with the uncertainty, just not knowing.”* (Female, 40 years old)

The long and uncertain patient journey from the moment they notice their symptoms up to the referral, has its emotional toll visible during their first visit to the medical specialist. As one HCP observed:

“*What we notice ourselves is that when someone comes to their first outpatient visit, we're already way behind in the hospital because everything has taken so long. While we're basically just getting started. And on the patient's side, all their patience is already gone, even though we haven't done anything yet here. So that's just because the uncertainty has lasted so long, which obviously causes such a big problem*.” (Female, 40 years old)

Although the diagnostic trajectory can be complex and fragmented, many HCPs noted the strong collaboration across medical specialities. Multidisciplinary consultations play an important role:

“*Yes, in that sense, it's very efficient. If you're sitting there with twenty people, then there are twenty people thinking about you. And even if it's only for five minutes, that still adds up to a hundred minutes of specialist time.”* (Male, 43 years old)

While the number of available specialists in rare cancer care is often limited, several HCPs noted that this can be advantageous. Rare cancer pathways are frequently managed by smaller, well-connected teams, which may foster more effective interdisciplinary collaboration and facilitate direct communication. As one HCP explained:

“*It sounds a bit strange, but sometimes one advantage of a rare cancer is that the teams are very small, so the lines of communication between the different specialties are short, so to speak.”* (Female, 51 years old)

Given the delays and complexity, managing expectations early is essential, as several HCPs emphasized. From the first hospital visit, patients are informed that diagnosis can take time, which is especially frustrating for those already referred from elsewhere. As one HCP explained:

“*People say, ‘I've been at this for months and still don't know what it is.' We try to explain we're often starting again from zero. That can be hard for patients and lead to frustration or even emotional distress.”* (Female, 40 years old)

Another HCP added:

“*Yes, so providing process information, explaining in advance that it's going to take time before the diagnosis is fully established. What steps are usually involved, and when someone will get the full picture. (…) Explain for example that ‘We're going to do this scan, then a biopsy, and the pathologist needs some time to assess that'.”* (Male, 47 years old)

Many HCPs stressed that when clear guidelines or care pathways are lacking, decision-making must carefully balance medical evidence with the patient's individual needs and preferences to offer the best care. As one HCP explained:

“*So, on the one hand, you're really missing solid evidence, a guideline or a care pathway, and on the other hand, patient-related factors can also influence the decision you actually want to make. Of course, we will still see and discuss the patient, but then, yes, it always ends up being a bit of a mix: what is the medical advice, what can the patient handle, and what does the patient want. So yes, that sometimes makes things open to multiple interpretations, so to speak*.” (Female, 44 years old)

Finally, emotional support and open dialogue with the patient are essential, as many HCPs stated, especially during waiting periods:

“*It's not just about giving information, but also asking what can help you or what you're worried about. What can I do to support you? Every patient is different. I also usually ask if they have someone to accompany them to appointments or when receiving results. Making it a topic of discussion, how are you doing while waiting, what do you need, or what would help, is really important.”* (Female, 44 years old)

## Striving for precision: the paradox of reaching a diagnosis

The difference between primary and secondary care is apparent when considering how rare cancers are managed. As one HCP stated:

“*From the hospital's perspective, a medical oncologist or surgeon sees many rare cases within their specialty. They often think: ‘how could this have been missed?' But that perspective comes from someone who frequently encounters the diagnosis, it requires a different way of thinking.”* (Female, 46 years old)

As such, experience plays a key role in managing rare cancers. As one HCP noted:

“*Once you start collecting rare cancers, they become less rare on your desk, and less difficult to deal with.”* (Male, 54 years old)

Many HCPs said that the impact of a delayed diagnosis on clinical and rehabilitation outcomes depends on the type of tumor and the length of the delay. Moreover, they emphasized that, for patients, even a short wait can feel distressing:

“*Yes, and of course, even if someone is seen within two weeks, it can still feel, especially if something malignant is found, like those two weeks were far too long. But fortunately, most tumours grow very slowly, over the course of years, so in terms of the size of the tumor or how treatable it is, those two weeks usually don't make a real difference. Still, it feels that way like you could have started treatment two weeks earlier if only you had known sooner.”* (Female 44, years old)

To reach the correct diagnosis, multiple HCPs emphasized the central role of imaging and pathology. As one HCP stated, using figurative language to highlight the authority of pathology in the diagnostic process:

“*The pathologist is the boss in my job. He says what it is.” (Male, 64 years old)*.

Another HCP said:

“*So, we really have to rely on imaging analysis, unless you have tissue diagnostics.”* (Female, 62 years old)

When HCPs were asked what effect diagnostic delay has on them, many stated that it is more devastating for the patient than for themselves. As one HCP said:

“*I feel worse for the patient than for myself.”* (Female, 38 years old)

HCPs also highlighted how frustrating it can be when system-level barriers delay diagnosis. Despite their desire to act quickly, especially in urgent situations, HCPs often found themselves navigating bureaucratic obstacles, making numerous phone calls, and coordinating across departments. These tasks are often incorporated into demanding and time-constrained schedules, causing delays that can frustrate both HCPs and patients. As one HCP put it:

“*You want to do your best for the patient, but you're blocked in all sorts of ways. It has to be done between patients, and that causes delays. Other patients get frustrated because the clinic is running behind.”* (Male, 49 years old)

HCPs expressed difficulty in providing psychosocial support during the diagnostic phase, particularly when patients lack a clear diagnostic group to relate to. As one explained:

“*It's quite difficult, because in a diagnostic process you don't necessarily have a diagnosis yet. So people don't know which group they belong to.”* (Female, 40 years old)

The same HCP elaborated on the challenges of offering support at this stage:

“*At this point, it's not really relevant yet, because there's no diagnosis. I can imagine some people might have a need, but maybe not, if you don't know whether you have cancer, for example, joining a peer group of people who do can feel premature. That makes it really difficult to address.”* (Female, 40 years old)

Another HCP noted that it may be too early for psychosocial support:

“*During the diagnostic process itself, once they're with us, it's not a very long period or anything. So, it's not like we're already saying: you need to contact social work or a psychologist. That's still too early at that point.”* (Female, 44 years old)

However, it was mentioned that there is psychological help that can be offered from the start of the trajectory, and that having a fixed point of contact can make a significant difference in recognizing and responding to emotional needs early on:

“*And at first, you notice that people are a bit skeptical about it, they don't all immediately say, ‘Oh great, I'd like that'. But actually, we can refer them quite easily, and by having one's own delegated professional, so to speak, the moment someone becomes psychologically in distress, they can get in touch, and we can then refer them on.”* (Female, 51 years old)

Several HCPs highlighted the value of patients being accompanied by someone from their social environment. Patients may not always express their symptoms or struggles clearly, whereas a partner or family member can often provide additional information. As one HCP stated:

“*I always appreciate it when someone comes along to the consultation, because then you're more likely to hear what's really going on. If a patient finds it hard to put things into words, sometimes a partner or daughter might say, ‘But remember that time you had that really intense pain attack?' Then the patient goes, ‘Oh yes.' That way, it comes up in the conversation.”* (Female, 44 years old).

According to some HCPs, reaching a diagnosis presents a paradox: it often means lifting patients out of uncertainty, which can be more distressing than receiving difficult news itself:

“*(…) uncertainty is often even worse than the bad news itself.”* (Male, 54 years old)

While speeding up the diagnostic process may not significantly impact the disease trajectory, some HCPs emphasized that reducing the period of uncertainty is crucial for patients' emotional wellbeing. As one HCP explained:

“*You can bend over backwards to try and shorten the diagnostic process by a few days, but in terms of the course of the disease, that really means absolutely nothing. And you really have to realize that clearly, how much effort and energy you're putting into it. That said, what I do think is important is the period of uncertainty regarding the diagnosis, you really do want to keep that as short as possible for the well-being of the patient.”* (Male, 47 years old)

## Discussion

### Main findings

According to HCPs, the diagnostic process for rare cancers is often hindered by vague and non-specific symptoms. HCPs rely on time, clinical intuition, and safety-netting to navigate uncertainty, while avoiding mis-, under- and overdiagnosing serious diseases like rare cancers. Clear communication and managing expectations with patients are seen as essential. Once cancer is suspected, the process usually accelerates, especially in academic hospitals. Integrated, collaborative teams and multidisciplinary input help when clear pathways are lacking. Apart of the possible effect on clinical and rehabilitation outcomes, the emotional strain of delay on patients is significant. Psychosocial support is hard to offer early due to diagnostic uncertainty, although HCPs consider emotional check-ins, providing a fixed point of contact and encouraging patients to bring someone to consultations as helpful. Ultimately, minimizing the period of uncertainty is key to the overall wellbeing of the patient.

### Interpretation of the findings

In our study, we found that rare cancers may present with vague, gradually progressing symptoms that could closely resemble those of common benign conditions. GPs reported prioritizing more common diagnoses, citing the low probability of encountering a rare cancer in routine practice. Medical education traditionally encourages HCPs to “think of horses when hearing hoofbeats,” meaning they consider common conditions before rare ones (Boening and Burger, 2018). Nevertheless, GPs must remain alert to the possibility that a “zebra” (i.e., a rare condition)may be the underlying cause. This diagnostic uncertainty, combined with the tendency to attribute symptoms to more prevalent illnesses, poses a substantial challenge in general practice and represents a key barrier to the timely diagnosis of rare cancers where symptoms resemble common, self-resolving diseases (Moore et al., 2024). This difficulty is further compounded by the very low incidence of rare cancers (Gatta et al., 2011), and explains that an individual GP is statistically unlikely to encounter such cases frequently in their career.

Overall, our findings illustrate how HCPs continually navigate competing diagnostic risks. They aim to limit the risk of missed or delayed diagnosis while also limiting overdiagnosis, misdiagnosis, avoidable patient distress, and additional pressure on the healthcare system. Balancing these risks highlights the tensions in making diagnostic decisions when symptoms are atypical and resources constrained. This interpretation aligns with a qualitative primary care study identifying ‘managing risk' as central to cancer presentation, detection, and referral, reflecting tensions between diagnostic uncertainty, fragmented care, and organizational pressures (Cook et al., 2014). While aiming for a timely diagnosis, GPs deliberately use *time* as a diagnostic tool to observe symptom progression, striving to balance early detection with the avoidance of unnecessary tests. This approach, however, can lead to mis- and underdiagnosing. Irving and Holden (2013) supported this by showing that while the time-efficiency principle refers to using time purposefully to improve diagnostic accuracy in primary care, it also carries the risk of delayed or missed diagnoses when symptom trajectories are unclear (Irving and Holden, 2013). This proposes the question on how to make optimal use of time as a diagnostic tool while limiting mis- or not diagnosing.

GPs in our study often reported relying on *intuition*, commonly described as “a gut feeling,” and vigilance when symptoms persist without clear explanation. This is in line with a systematic review, in which the authors reported that GPs' “gut feelings,” a rapid, experience-informed intuition integrating multiple verbal and non-verbal cues, were associated with over a fourfold increase in cancer diagnosis. These intuitive judgments became more reliable with greater clinical experience and patient familiarity (Smith et al., 2020).

This range of verbal and non-verbal cues not only informs clinical judgment, but also underpins the provision of patient support through *safety-netting*, an essential strategy for managing uncertainty that depends on effective communication and patient follow-up (Ayodapo et al., 2023). A recent study on safety-netting in patients with lung cancer showed that patients prefer active safety-netting over passive safety-netting, with the former being supported by comprehensive consultation practices and a clearly communicated diagnostic plan, and the latter potentially leading to negative responses or reduced confidence in care (Black et al., 2022). Nevertheless, while safety-netting can enhance patient reassurance and trust, it may inadvertently increase pressure on healthcare systems by generating more follow-up consultations and diagnostic procedures, particularly when patients are encouraged to return even for mild or ambiguous symptoms. This is supported by a qualitative interview study with GPs, in which the authors found that although GPs generally support safety-netting, they are unconvinced that key guideline elements, e.g., informing patients of all (including negative) results, flagging recurrent unexplained symptoms, and assigning specific follow-up responsibility, are feasible given current staffing and time pressures in general practice (Tompson et al., 2019).

Structural issues within healthcare systems, such as lack of coordinated care pathways for rare cancers, contribute to fragmented care and impact overall patient experience (De Heus et al., 2021). Nonetheless, in our study, we found that once cancer is suspected, the diagnostic process typically *accelerates*, especially within academic medical centers. This can be attributed to the fact that such centers possess specialized expertise, advanced resources, and multidisciplinary teams that enable more efficient and timely diagnosis and treatment (Tumiene and Graessner, 2021). However, HCPs in our study explained that logistical “delays” remain, such as waiting times for MRIs. These waiting times, which can vary between a few days and up to 2 weeks (Dutch Healthcare Authority, 2026), highlight the complexity of further accelerating the diagnostic process. While some may consider these timeframes relatively short, they can still contribute to patients' anxiety, perceived delay, and challenges in maintaining functional independence. It raises the question of whether accelerating diagnostic pathways could also facilitate timely initiation of rehabilitation services, specifically for those with a rare cancer, and if this is even possible within current system constraints.

While many HCPs in our study stated that diagnostic delays rarely affect clinical outcomes, they emphasized the notable *psychological impact* such delays can have. Importantly, research by Padilla et al. in the Netherlands confirmed that prolonged diagnostic trajectories are common among patients with a rare cancer, varying form less than three months to more than 12 months, and are frequently accompanied by initial misdiagnoses and incremental delays in referral and investigation (Padilla et al., 2024). This pattern is observed alongside survival disparities: patients with a rare cancer experience poorer outcomes compared to those with a common cancer (De Heus et al., 2022). Although a direct causal link between delay and survival remains complex, the consistent association reinforces the importance of minimizing delays to improve both psychological wellbeing and, potentially, clinical and rehabilitation outcomes. Prolonged time until hospital referral often leads to significant emotional distress for patients, largely due to extended periods of diagnostic uncertainty. As noted by many interviewed HCPs, uncertainty is a persistent and influential factor throughout the diagnostic journey, particularly for patients with a rare cancer. To manage this uncertainty and reduce frustration from patients, they viewed clear, honest communication as essential. Early expectation management, including transparent information about probability, ambiguity, and the complexity and potential length of the pathway, was seen as critical to supporting patients. This is supported by Meyer et al., stating that uncertainty should be acknowledged openly and communicated clearly with patients, families, and healthcare teams (Meyer et al., 2021). Importantly, communication about uncertainty involves more than merely explaining possible diagnosis and diagnostic steps. It requires HCPs to also acknowledging what remains unknown, supporting patients in making sense of an evolving pathways, and providing clear follow-up and possible re-evaluation, relying on specific communication skills (Medendorp et al., 2021). However, Smith et al. found in a systematic review and meta-analysis that diagnostic uncertainty was found to be common in primary care, yet rarely communicated explicitly to patients (Smith et al., 2020).

According to HCPs from secondary care, multidisciplinary collaboration in rare cancer care is generally robust and efficient, especially within small, specialized teams. A recent study by the European Reference Network for adult rare solid cancers (ERN EURACAN) supported this, noting that patients are typically referred to expert Multidisciplinary Team Boards (MDTBs), organized locally or regionally (Blay et al., 2024). These MDTBs operate with notable speed and structure, often reviewing cases within minutes during weekly meetings, while *striving for precision*. EURACAN emphasizes that this streamlined process is a deliberate feature of highly efficient teams (Blay et al., 2024), and an essential asset for delivering timely, accurate, expert care for patients with a rare cancer.

Regarding psychosocial support, HCPs said that offering it in the pre-diagnostic phase may be premature. Although little is known about psychological support pre-diagnosis, Berman et al. stated that providing supportive care throughout the diagnostic pathway is crucial, because it can help patients with managing emotional distress, uncertainty, and anxiety (Berman et al., 2020). Building on this, Dekker et al. argue that distinguishing between normal, adaptive emotional responses, which are typical stress reactions that help patients cope, and maladaptive ones, which interfere with daily functioning, is important, as professional support is primarily needed when patients exhibit maladaptive responses (Dekker et al., 2017).

Finally, while the term “delay” has been used in this study in relation to hurdles throughout the diagnostic pathway, HCPs responded critically to its use, suggesting the term should be applied with care. From their perspective, they act based on professional expertise within the constraints of the healthcare system. Obviously, none of the HCPs would intentionally prolong a patient's diagnostic trajectory. However, they acknowledged that certain factors within the trajectory might inadvertently impact progress. This raises important questions about whether “delay” is the most accurate term, or if it would be better to consider the diagnostic process as a “prolonged trajectory,” influenced by various factors. Rather than merely labeling a diagnostic trajectory as “delayed,” our findings highlighted the importance of examining the factors that shape its duration and identifying ways to accelerate it, where feasible. Moreover, it is worth considering whether accelerating the trajectory is always required, or if the emphasis should be on optimizing the quality and appropriateness of the diagnostic process and how to support patients.

## Strengths and limitations

To our knowledge, this is the first study to explore HCPs' perspectives on the diagnostic trajectory of patients with a rare cancer. We aimed to capture the full diagnostic trajectory, and deliberately included a heterogeneous group of HCPs, from various specialties such as general practice, pathology, and surgical oncology. This diversity provides a broad range of professional insights into the diagnostic process of rare cancers. However, the heterogeneity of the sample may also be a limitation. Specifically, differences in professional roles and contexts could lead to variation in how the diagnostic process is perceived, and the limited number of participants per specialty may reduce the depth and representativeness of each perspective. Moreover, recruitment through professional networks and social media may have introduced selection bias, potentially limiting the diversity of perspectives, and affecting the transferability of the findings. Although the study sample included HCPs from various specialties, it lacked explicit attention to diversity in practice settings, as most participants were based in academic hospitals, likely reflecting highly specialized environment with concentrated rare cancer expertise, lower diagnostic delay, and stronger collaboration infrastructues. This limits the generalizability of these findings to peripheral hospitals and primary care. Additionally, while this study focuses on informing patient support strategies, it reflects only the perspectives of HCPs. Therefore, in a parallel study, we explored the experiences of patients with a rare cancer regarding diagnostic delay. Another limitation is that GPs encounter very few patients with a rare cancer, which may result in their responses being shaped by isolated or anecdotal experiences. Finally, although online interviews have become common practice since the COVID-19 pandemic, this mode of data collection may have limited the depth and richness of participants' responses.

## Implications for research and practice

There is an urgent need to explore the psychological impact of diagnostic uncertainty on patients with a rare cancer. Future research should investigate the optimal timing and forms of psychosocial support during the diagnostic pathway, and options for integration across both primary and secondary care settings. This should also encompass broader concepts, such as overall patient empowerment, self-management, coping strategies, and resilience-building, which are essential for helping patients navigate uncertainty. Additionally, an area for further investigation relates to how HCPs support patients in navigating diagnostic uncertainty. This includes managing patients' uncertainty, fostering resilience in patients, and providing various types of support, such as facilitating social networks, offering empathy, or delivering practical guidance. It is important to examine how these supportive approaches vary across different care settings. Early engagement with rehabilitation professionals may help patients maintain functional independence, manage physical and psychosocial challenges, and prepare for treatment and recovery. Future research should examine how rehabilitation interventions can be integrated early in the patient trajectory, alongside psychosocial support, to optimize patient participation in daily activities and work, and to enhance their overall QoL. Understanding how rehabilitation needs differ across primary and secondary care settings could inform patient-centered care strategies that preserve function while diagnostic uncertainty is being resolved. Insights into these contextual differences could inform the development of patient-centered strategies for managing care during uncertain or transitional phases.

Further, a clear and consistent definition of “diagnostic delay” and criteria for when such delays become clinically significant are currently lacking. To accurately assess the impact of diagnostic delay on both clinical and psychosocial outcomes, future research should focus on comparing diagnostic trajectories within specific rare cancer types. For example, studying patients with sarcoma from symptom onset through confirmed diagnosis may help identify critical points where delays are most likely and elucidate contributing factors. Future research should also evaluate different models of safety-netting and follow-up care. While efforts to improve safety-netting may increase the number of follow-up appointments and contribute to higher workloads in general practice, it is necessary to explore which follow-up strategies, whether in-person visits, telephone contacts, or digital tools, are both clinically effective and operationally sustainable. Also, understanding how patients interpret follow-up intervals, particularly when they perceive them as delays, and how communication can better align patient and professional expectations is essential for improving both the diagnostic experience and health system efficiency.

HCPs should proactively manage patient expectations from the outset, clearly acknowledging the absence of standardized diagnostic pathways for many rare cancers. Strengthening two-way, patient-centered communication is critical, not only for effective information exchange, but also for recognizing and addressing patient concerns and emotional needs throughout the diagnostic process (Gilligan et al., 2017). When knowledge is limited and resources are scarce, it is important to communicate this transparently with patients and involve them in the shared decision-making process, helping them understand what they can expect, what is possible, taking patient preferences into account (Braddock, 2013).

Providing patients with delegated professional and sustained emotional support is essential to help them feel informed, supported, and engaged, even amid ongoing diagnostic uncertainty (Katerenchuk and Salas, 2023). This continuity can foster trust and resilience, contributing to better patient experiences during this challenging phase (King et al., 2008). Healthcare systems and professionals should also consider the operational implications of enhanced safety-netting. Structuring follow-up appointments efficiently, whether by in-person, telephone, or digital means, must balance patient needs with the realities of clinical workload and resource constraints.

## Conclusion

Rare cancer diagnoses are often delayed due to vague symptoms and uncertainty. HCPs rely on clinical judgment, safety-netting, and clear communication to manage this. Although diagnosis speeds up once cancer is suspected, systemic delays and emotional strain remain and can affect patients' participation in daily life and rehabilitation activities, and reduce their overall QoL. Early psychosocial support is both challenging and essential, with providing a fixed point of contact and emotional check-ins helping patients cope. Future research should define diagnostic delay, assess the impact, and explore optimal support strategies.

## Data Availability

The datasets presented in this article are not readily available due to privacy or ethical restrictions. Requests to access the datasets should be directed to s.duijts@iknl.nl.
