# Supplementary material for: Healthcare professionals' perspectives on diagnostic delay in rare cancers and options for patient support: a qualitative interview study
Source: Front Psychol. 2026 Oct 9;17:1945741. doi: 10.3389/fpsyg.2026.1945741 (PMC13749395; doi:10.3389/fpsyg.2026.1945741)
Supplement: Supplementary file 1 [file Supplementary_file_1.pdf]

Supplement 1. Quotes

Theme	Subtheme	Quote
Theme 1	Diagnostic trajectory	*"They are often very vague and difficult to pinpoint symptoms. Like a stuffy nose, for example, well, everyone gets that from time to time."* (Male, 38 years old)<br><br>*"Let me emphasize that, in the early stages of symptoms in someone with a brain tumor, things are often very unclear, very vague, vague symptoms. People often think, 'Oh yeah, a brain tumor always starts with headaches', but that's not the case. It can be very subtle things: somewhat forgetful, maybe burned out, overworked, depressed, which in hindsight, turn out to have been the first signs of a brain tumor."* (Male, 49 years old)<br><br>*"The point is that general practioners are well trained in recognizing what is normal, what a normal flu looks like, what a normal cold looks like."* (Male, 41 years old)<br><br>*"As a general practitioner, you are constantly assessing probabilities and risks, and rare diagnoses are always somewhere on your list, but usually very low. The prior probability is typically very small. So, before the patient even walks in, the likelihood is already low, and even after hearing the patient's story, it often remains low. I think it is good practice not to immediately pursue extensive diagnostics."* (Female, 44 years old)<br><br>*"If we were to diagnose everyone very quickly and run extensive tests on everyone, we might miss fewer tumors, or perhaps none at all, but we'd also end up doing a lot of unnecessary things. This daily risk assessment is part of our profession, and inevidebly, the downside is that we are going to miss things as well."* (Female, 46 years old)<br><br>"You don't want all those people to go to the hospital. That puts so much pressure on your scans and your x-rays and whatever. Overdiagnosis for nothing is not good" (Male, 64 years old)<br><br>"I think it's not just theoretical, but also practical and financial, right? You might want to order fifty scans for every patient when things aren't immediately clear, but that's unaffordable and unreasonable. So, I think you need to prioritize, based on logistics, based on prior probability, based on... well, that's also good medicine." (Male, 43 years old)<br><br>*"There is certainly a delay involved, which is partly intentional, as we consciously use time as a diagnostic tool."* (Male, 41 years old)<br><br>"As general practitioners, I believe we truly use time as a diagnostic criterion" (Female, 44 years old)
	Experiences HCP	*"I still quite often find myself giving what's called safety-netting advice; telling patients to return if their symptoms haven't resolved within three weeks."* (Female, 44 years old)<br><br>*"So no, sometimes it's still about following up on that kind of advice, and of course that depends both on how you communicate it, how the person takes it in, and also on who they are and how life unfolds."* (Female, 44 years old)
	Patient support	*"It's a balance between being honest, like saying, 'this doesn't sit well with me', or 'I don't fully trust it', or 'I'm a bit concerned', and at the same time not unnecessarily frightening the patient."* (Female, 46 years old)

		"Yes, I think clarity is crucial, really taking the time to explain why certain steps are taken. For patients, it can be very difficult to understand that, even in this day and age, we don't immediately know exactly what's going on or what the plan will be. I believe it's important to take the time to explain that clearly, be honest about the current gaps in knowledge or what is still needed, and to provide a realistic timeline. Don't say, 'We'll do a biopsy today and have the results tomorrow', if that's not the case, because we won't." (Female, 44 years old)<br><br>"I think it's important to communicate effectively with your patients, to show genuine involvement, and to offer support. Together, you assess what is needed. The psychosocial aspect is crucial and something you should always monitor closely during that period" (Female, 44 years old)<br><br>"If someone comes to talk, you must give them your full attention. So don't get distracted by a phone or people walking in. Make sure you have all the available information about the patient clearly in your mind, so you don't make any mistakes." (Female, 46 years old)<br><br>"And furthermore, I think it's very important in communication to be clear and straightforward, so that the patient knows what to expect." (Female, 46 years old)<br><br>"So, communication remains especially important. And if you look at it from the perspective of a general practice, I also think it's simply important to communicate well with your patients, to show involvement, and to offer support. And then you look together with people at what is needed, yes. And that the psychosocial aspect is the most important part, something you always need to keep a close eye on during that period." (Female, 36 years old)<br><br>*"Continue to heed your 'something-is-not-right' sense and remain vigilant as a physician: when the clinical picture does not fit, reflect and act accordingly. Stay alert if a patient returns several times without a clear explanation and intervene as needed."* (Female, 36 years old)
Theme 2	Diagnostic trajectory	*"No, that does fall under the responsibility of the general practitioner, to make a proper plan and ensure that the referral is appropriate.''* (Male, 41 years old)<br><br>*"And so, it's inherently a more difficult population, because it's not a homogeneous group. We're dealing with gaps in our knowledge, and we have to make do with knowledge of lower quality."* (Male, 54 years old)<br><br>"Look, I'm in an academic hospital, so by the time they get to us, it's already being investigated. But they've often had a longer lead-up in the peripheral hospital, so indeed, it may have taken relatively long before it was considered." (Male, 43 years old)<br><br>"No, it's not that bad. But I work in an academic hospital, so I think delays are minimal there, and that makes things relatively easy for us, because people are referred to us when things are unclear. Then the diagnostics are started quite quickly, because you're already heading in a certain direction. I think it's much more difficult in a peripheral hospital." (Female, 44 years old)
	Experiences HCP	*"Sometimes you have to work with the tools you have and navigate the uncertainties, and that's sometimes the art of medicine: figuring out how*

		to ensure as swift and smooth a diagnostic process as possible." (Female, 62 years old)<br><br>*"Look, the moment we as general practitioners think this might be cancer, things usually move quickly."* (Female, 46 years old)<br><br>*"If you work in an academic hospital and you see more of those rare pathologies, you're able to recognize them more easily. And that's not at all a criticism of those who mainly see common conditions, they're often much better at spotting those presentations. But for rare cases, it can lead to delays. The likelihood of a disease influences how thorough your diagnostics are. But it's not just about seeing it, you have to recognize it in the patient's story."* (Male, 43 years old)<br><br>*"People prefer to know sooner where they stand psychologically, so I notice that they struggle with the uncertainty, just not knowing."* (Female, 40 years old)<br><br>"Well, I think uncertainty is one of the most difficult emotions a patient can experience. So, you want clarity about what you have as soon as possible." (Male, 47 years old)<br><br>"It's the worst part of the entire treatment process, because it's all about uncertainty." (Male, 72 years old)<br><br>*"What we notice ourselves is that when someone comes to their first outpatient visit, we're already way behind in the hospital because everything has taken so long. While we're basically just getting started. And on the patient's side, all their patience is already gone, even though we haven't done anything here yet. So that's just because the uncertainty has lasted so long, which obviously causes such a big problem."* (Female, 40 years old)<br><br>*"It sounds a bit strange, but sometimes one advantage of a rare cancer is that the teams are still very small, so the lines of communication between the different specialties are short, so to speak."* (Female, 51 years old)<br><br>"Because it's something very rare the people involved form a closer-knit team than if you had twenty people working on it. So that naturally makes collaboration easier." (Female, 40 years old)<br><br>"It's a small team, and because it's such a rare type of cancer, those teams also have good contact with each other worldwide." (Female, 51 years old)
	Patient support	*"Yes, in that sense, it's very efficient, if you're sitting there with twenty people, then there are twenty people thinking about you. And even if it's only for five minutes, that still adds up to a hundred minutes of specialist time."* (Male, 43 years old)<br><br>*"People say, 'I've been at this for months and still don't know what it is.' We try to explain we're often starting again from zero. That can be hard for patients and lead to frustration or even emotional distress."* (Female, 40 years old)<br><br>*"Yes, so providing process information, explaining in advance that it's going to take time before the diagnosis is fully established. What steps are usually involved, and when someone will get the full picture. (...) "Explaining that 'We're going to do this scan, then a biopsy, and the pathologist needs some time to assess that'."* (Male 47 years old)

		"And that patients are also kept informed when a result takes a long time, why that is. That they're told, for example, 'we're dealing with a tumor we don't see every day, and it requires further investigation.' If you explain that and stay in dialogue with the patient, involve them in the process, that's something you have to do. You can't just say, 'we'll call you in four weeks with the results.' That's not acceptable." (Male, 65 years old)<br><br>*"So, on the one hand, you're really missing solid evidence, a guideline or a care pathway, and on the other hand, patient-related factors can also influence the decision you actually want to make. Of course, we will still see and discuss the patient, but then, yes, it always ends up being a bit of a mix: what is the medical advice, what can the patient handle, and what does the patient want. So yes, that sometimes makes things open to multiple interpretations, so to speak."* (Female, 44 years old)<br><br>*"It's not just about giving information, but also asking what can help you or what you're worried about. What can I do to support you? Every patient is different. I also usually ask if they have someone to accompany them to appointments or when receiving results. Making it a topic of discussion, how are you doing while waiting, what do you need, or what would help, is really important."* (Female, 44 years old)
Theme 3	Diagnostic trajectory	*"Once you start collecting rare cancers, they become a little less rare on your desk, and a little less difficult to deal with."* (Male, 54 years old)<br><br>*"The pathologist is the boss in my job. He says what it is". (Male, 64 years old)*<br><br>*"So, we really have to rely on imaging analysis, unless you have tissue diagnostics."* (Female, 62 years old)<br><br>"Well, diagnostics have improved tremendously, and that's very important for rare tumors. So, to summarize: pathology is the cornerstone of a proper diagnosis and effective treatment." (Male, 72 years old)<br><br>"That really is something of a completely different order. And it wasn't that long ago. With that, we've also reminded ourselves that we really need to hold back, if we don't have a definitive pathology result, we must remain very cautious and not too quickly assume it will be this or that and proceed in that direction. Because it's also difficult when you must completely retract a diagnosis you had considered. And that does happen a few times a year, and it's related to waiting for that PA result. People still want to hear something, and we think we can already interpret it. But that's something you really shouldn't do when it comes to rare conditions." (Male, 65 years old)
	Experiences HCP	*"From the hospital's perspective, a medical oncologist or surgeon sees many rare cases within their specialty. They often think: 'how could this have been missed?' But that perspective comes from someone who frequently encounters the diagnosis, it requires a different way of thinking."* (Female 46 years old)<br><br>*"Yes, and of course, even if someone is seen within two weeks, it can still feel, especially if something malignant is found, like those two weeks were far too long. But fortunately, most tumours grow very slowly, over the course of years, so in terms of the size of the tumor or how treatable it is, those two weeks usually don't make a real difference. Still, it feels that way like you could have started treatment two weeks earlier if only you had known sooner."* (Female 44, years old)

		“I feel worse for the patient than for myself.” (Female, 38 years old)<br><br>"So, if it’s unclear, it doesn’t really affect my well-being, but yes, it is annoying. But especially for the patient, that’s really what it’s about." (Female, 44 years old)<br><br>*“You want to do your best for the patient, but you’re blocked in all sorts of ways… It has to be done between patients, and that causes delays. Other patients get frustrated because the clinic is running behind.”* (Male, 49 years old)<br><br>*“I always appreciate it when someone comes along to the consultation, because then you’re more likely to hear what’s really going on. If a patient finds it hard to put things into words, sometimes a partner or daughter might say, ‘But remember that time you had that really intense pain attack?’ Then the patient goes, ‘Oh yes.’ That way, it comes up in the conversation.”* (Female, 44 years old).
	Patient support	*“It’s quite difficult, because in a diagnostic process you don’t necessarily have a diagnosis yet. So people don’t know which group they belong to.”* (Female, 40 years old)<br><br>“*At this point, it’s not really relevant yet, because there’s no diagnosis. I can imagine some people might have a need, but maybe not, if you don’t know whether you have cancer, for example, joining a peer group of people who do can feel premature. That makes it really difficult to address.”* (Female, 40 years old)<br><br>*“During the diagnostic process itself, once they’re with us, it’s not a very long period or anything. So, it’s not like we’re already saying: you need to contact social work or a psychologist. That’s still too early at that point.”* (Female, 44 years old)<br><br>*“And at first, you notice that people are a bit skeptical about it, they don't all immediately say, 'Oh great, I’d like that’. But actually, we can refer them quite easily, and by having one’s own delegated professional, so to speak, the moment someone becomes psychologically in distress, they can get in touch, and we can then refer them on.” (Female, 51 years old)*<br><br>"Yes, if people have someone they can bring along, that’s helpful, because then we can also inform that person and ask them to think along with us." (Female, 54 years old)<br><br>*“(…) uncertainty is often even worse than the bad news itself.”* (Male, 54 years old)<br><br>*You can bend over backwards to try and shorten the diagnostic process by a few days, but in terms of the course of the disease, that really means absolutely nothing. And you really have to realize that clearly, how much effort and energy you're putting into it. That said, what I do think is important is that the period of uncertainty regarding the diagnosis, you really do want to keep that as short as possible for the well-being of the patient."* (Male, 47 years old)

* Note: quotations in italics are part of the reported results; all other quotes serve a supplementary purpose.

Supplement 2. Interviewguide

Topic	Content
Introduction	Hello, my name is (name). I am a Health Sciences student at (name university). This study is part of my internship at (name organization). Next to me is (name), also student Health Sciences. She is conducting parallel research on the experiences and perspectives of **patients** regarding diagnostic delays in rare forms of cancer. (name) is present to provide practical support and take notes.
Aim of the study	The aim of this study is to explore the perspectives and experiences of healthcare professionals regarding the diagnostic trajectory of rare forms of cancer. We also want to identify possible ways to support patients with a rare cancer during this process.
Information prior to the interview	Prior to this interview, you received an email (or: envelope by post) containing detailed information about this study and a consent form. I have received your signed consent form. Thank you for that. Do you have any questions or comments about the documents you received?
Duration of the interview	The interview will take approximately 45 minutes.
Experience	This interview is about your experiences. This means there are no wrong answers. However, if we stray too far from the topic, I will gently steer the conversation back. This does not mean I am not interested in what you have to say, but I would like to cover the questions I have prepared within the allotted time.
Anonymity & confidentiality	The interview will be recorded (show recorder), but you will remain anonymous. Details such as your name, hospital, etc. will not be transcribed. After the study is completed, this recording will be deleted.
Quitting interview	You may stop the interview at any time, and you may also indicate if you prefer not to answer a question.
Form of address	Do you prefer that I address you formally (using "you") or may I use the informal form? (In Dutch)
Start interview	Do you have any questions or comments so far? If not, I will start the recording now and we will begin the interview.

Topic	Content/ questions
Introduction (5 min)	(Keep the questionnaire nearby)<br><br>In the questionnaire, you indicated that you work as a (specialist / GP) in a .... hospital / primary care / practice, and that you are mainly involved in patients with…
Diagnosis rare cancer (5 min)	<u>Specialist:</u><br>Could you describe your <u>role</u> in the diagnostic trajectory of these patients? At what <u>point</u> in the trajectory do you typically see them? Could you share your <u>experiences</u> with the diagnostic trajectory of the patients you see (duration of the trajectory, delays encountered, if so, at which phase), either in general or by sharing a specific (or representative) example?<br><br><u>GP:</u><br>Rare cancers are seen less frequently in general practice compared to more common cancers. Could you describe your role in the diagnostic trajectory of a specific patient with a rare cancer? Could you share your experiences regarding the diagnostic trajectory for such patients (duration of the trajectory within primary care, what kind of symptoms prompt a referral, your insight into the different specialists the patient has seen)?

	<u>Both:</u><br>Are there any rare cancers where you feel delays in diagnosis are common and where the trajectory could potentially be made more efficient? Could you elaborate?
Diagnostic trajectory factors (15 min)	When it comes to factors that influence the diagnostic process: which ones do you consider to be important?<br><br>Can you indicate whether you think this factor could be **influenced** or **changed,** and **how**?<br><br>*Memory aid / prompts:*<br><br>• *Individual patient factors (emotions, etc.)*<br>• *Immediate social environment (e.g., social support network)*<br>• *Physician-related factors*<br>• *Healthcare system / politics / policy / guidelines*<br><br>*Overarching categories*<br><br>• *Patient factors*<br>• *Knowledge / awareness*<br>• *Healthcare system / policy / protocols*<br>• *Social factors*<br>• *Working conditions*<br>• *Patient–provider relationship*<br>• *Societal / political factors*<br>• *Accessibility / availability of information for referral to expert centers*<br><br>*Examples*<br><br>• *General awareness of rare cancers*<br>• *Education/training on rare cancers*<br>• *Having a long-standing relationship with the patient / continuity of care*<br>• *Consultation time / workload*<br>• *Access to appropriate diagnostic tests/resources*<br>• *Identifying the right specialist / expert center (knowledge/network)*<br>• *Availability of the appropriate specialist / expert center*<br>• *Patient factors: age, gender, education level, health literacy, emotions, coping style (e.g., avoidant: "if I don't think about it, it will go away"), knowledge, perceived SES, whether the provider realizes the patient doesn't understand, income (poverty/stress from other life issues)*<br>• *Social factors: caregiving responsibilities, obligations, work status*<br>• *Healthcare system: long waiting times due to staff shortages*<br>• *Society/policy: stigma related to certain cancers or patient groups. (It might be difficult to admit, but women are more often sent home with a diagnosis*

	of depression or burnout than men, only to later find that there was a physical cause after all.)<br><br>Are there any other factors we haven't discussed that you believe are important?<br>To what extent could they be changed or influenced, and how?
Time till diagnosis	Are there specific time intervals (e.g., from first symptoms to GP visit, from GP referral to hospital, or from the first to the second hospital) that, in your view, are particularly long and could potentially be shortened?<br><br>→ It's understandable if this is difficult to answer, as it varies by **tumor type**, and also because you work in **(fill in: primary / secondary care), so you may have less insight into what happens in (fill in: GP setting / hospital)**. But could you say something about this in general? Or perhaps share an example from your own perspective?
Wrong diagnosis (or long trajectory)	Have you ever encountered a situation where there was initially an 'all-clear' diagnosis *("everything looks fine"; no signs of cancer*) or a different diagnosis (*another condition*), and it later turned out to be a rare form of cancer?<br><br>If you have encountered a case like that (misdiagnosis or prolonged/difficult diagnostic process): What were the specific **turning points** in the process? Which steps, actions, tests, or information led to a correct diagnosis?
Impact delayed diagnosis	The diagnostic process can have a significant impact on patients (and their caregivers), for example in daily life, social functioning, work/finances, psychological/mental well-being, and overall quality of life.<br><br>In your view, what are the **psychological** and **social** effects on patients who go through a prolonged diagnostic process?<br><br>Do you think a delayed diagnosis affects treatment? If so, how?<br><br>Do you think a delayed diagnosis affects access to appropriate (follow-up) care (*e.g., specialized treatment, psychosocial support, or palliative care*)? If so, how?<br><br>Do you think a delayed diagnosis affects clinical outcomes? If so, how?<br><br>Have you encountered situations in your professional career where, thanks to actions you took, a faster diagnosis made a significant difference in the course of the disease?

Patient support	What could be done to better support patients during the diagnostic trajectory?<br><br>Who / which healthcare professionals play a role in that?<br><br>What is the role of the patient in that?<br><br>Does the patient's social environment play a role in this? If so, how?
Impact and opportunities for improvement in the healthcare provider/patient relationship and the physician/specialist relationship	What impact does diagnostic delay in rare cancers have on your professional practice and well-being? →How do you deal with that?<br><br>What is currently going well in the collaboration between GPs and specialists?<br><br>What could still be improved in the collaboration between GPs and specialists to optimize the diagnostic trajectory for rare cancers?
Closure	Those were all my questions.<br><br>Do you have any additions to what we just discussed? Are there any topics that haven't been covered or not addressed sufficiently?<br><br>Do you have any questions for me?<br><br>Then I would like to sincerely thank you for your participation in the interview and for sharing your experiences.<br><br>We will share the results of the study with you by email as soon as they are published.<br><br>If you have any further questions or comments, feel free to email me at (mailadress) or call me at (phonennumber).
